# Shifting Perceptions of Cosmetic Procedures: Rise of Medical Spas in Online Search Trends

**DOI:** 10.2196/83509

**Published:** 2026-07-15

**Authors:** Hong Ye, Sophia Yanjia Zhu, Britta Verhey, Joy Xu, Gordon Hyeonjin Bae

**Affiliations:** 1 Virginia Tech Carilion School of Medicine, Virginia Polytechnic Institute, Roanoke, VA, United States; 2 Department of Molecular, Cellular, and Developmental Biology, College of Letters and Sciences, University of California, Santa Barbara, Santa Barbara, CA, United States; 3 School of Medicine, University of Nevada, Reno, Reno, NV, United States; 4 David Geffen School of Medicine, University of California, Los Angeles, Los Angeles, CA, United States; 5 Department of Dermatology, Stanford School of Medicine, Stanford University, 450 Broadway Street, Redwood City, CA, 94063, United States, 1 650 441 2775

**Keywords:** patient safety, cosmetic procedures, aesthetic procedures, med spa, medical spa, nonphysician provider, dermatologist, online search trends, Google Trends

## Abstract

Our analysis of online search trends suggests that over the past decade, public search interest has increasingly associated certain aesthetic procedures, particularly injectables and laser hair removal, with medical spas rather than dermatologists.

## Introduction

In recent years, the rise of medical spas (med spas) has reshaped aesthetic dermatology. As cosmetic procedures are increasingly framed as wellness and self-care procedures, patients may view them as routine, driving demand for services performed by nonphysician providers. This raises concerns about patient safety and clinical oversight. Demand for cosmetic procedures continues to increase globally, with preferences varying by age, ethnicity, and geography [1]. Social media engagement has paralleled rising search interest in popular cosmetic terms, further shaping patient decision-making. To assess evolving public perceptions, we analyzed online search trends, comparing queries pairing common cosmetic procedures with either “med spa” or “dermatologist.”

## Methods

### Study Design

We used Google Trends (GT) to examine US relative search volumes (RSVs) from June 2015 to June 2025 for 7 aesthetic procedures—Botox injection, laser hair removal, chemical peels, laser skin resurfacing, microdermabrasion, microneedling, and dermal fillers. These procedures were selected to represent cosmetic treatments ranging from minimally invasive (eg, chemical peels) to more complex interventions (eg, laser skin resurfacing), spanning varying levels of procedural risk and required expertise. Search terms were identified via GT’s “Related queries” feature (filters: United States, all categories, web search) and supplemented by researcher consensus, capturing both generic descriptors and brand names. Each term was queried in combination with “med spa” or “dermatologist” to create matched pairs (Multimedia Appendix 1). Terms were excluded if GT returned RSVs of 0 for the majority of the study period.

For procedures with multiple search terms, monthly RSVs were averaged within each framing to produce a single composite time series. Paired 1-tailed *t* tests compared mean monthly RSVs between framings, with each month as a matched time point. Effect size was defined as the mean RSV difference (“med spa” minus “dermatologist”); positive values indicated higher RSVs for “med spa.” We reported 95% CIs. The normality of paired differences was assessed via the Shapiro-Wilk test; 3 procedures (Botox injection, microneedling, and lip fillers) showed departures from normality, and Wilcoxon signed rank tests confirmed all results. Statistical significance was set at *P*<.05.

### Ethical Considerations

This retrospective study is exempt from informed consent. It was a secondary analysis of existing data, it did not involve intervention or interaction with human participants, and the reviewed data were deidentified per the deidentification standard defined in Section §164.514(a) of the HIPAA (Health Insurance Portability and Accountability Act) Privacy Rule. The process by which the data were deidentified was attested to through a formal determination by a qualified expert, as defined in Section §164.514(b)(1) of the HIPAA Privacy Rule. This formal determination refreshed on December 2020.

## Results

**Figure 1.**
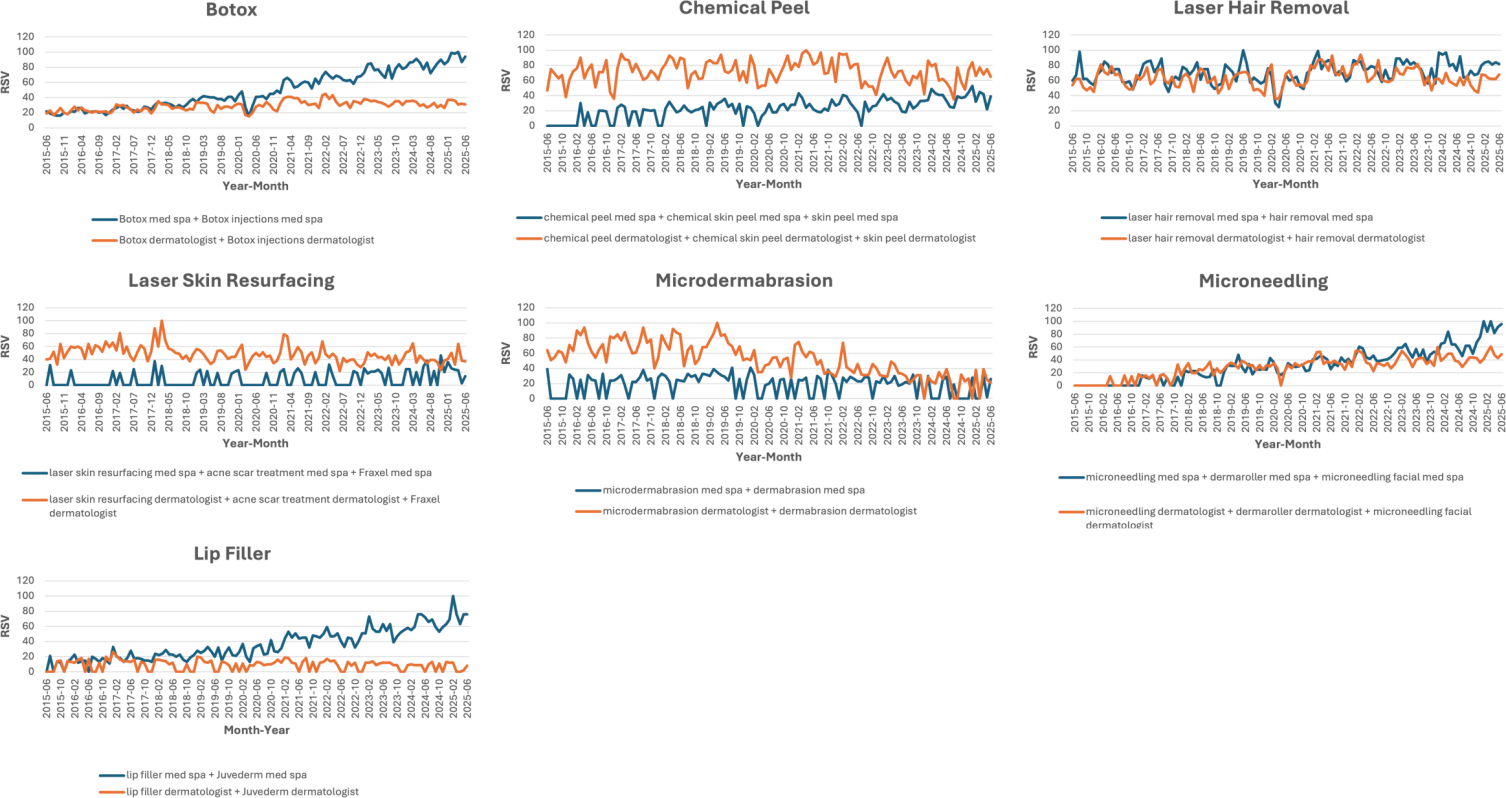
Relative search volumes (RSVs) over time for cosmetic procedures paired with “med spa” versus “dermatologist” (2015‐2025).

Significant differences in search interest emerged between “med spa” and “dermatologist” queries for all 7 procedures (Figure 1). “Med spa” framing was associated with significantly higher RSVs for lip fillers (“med spa”=35.7; “dermatologist”=9.1; effect size=26.6), Botox injection (“med spa”=49.4; “dermatologist”=29.1; effect size=20.3), laser hair removal (“med spa”=71.1; “dermatologist”=63.7; effect size=7.4), and microneedling (“med spa”=32.6; “dermatologist”=28.5; effect size=4.1) (Table 1). “Dermatologist” framing showed higher RSVs for chemical peels (“dermatologist”=71.6; “med spa”=22.8; effect size=−48.8), laser skin resurfacing (“dermatologist”=48.0; “med spa”=8.9; effect size=−39.1), and microdermabrasion (“dermatologist”=51.7; “med spa”=18.9; effect size=−32.8). All comparisons were statistically significant (all *P* values≤.004).

RSV is a normalized metric ranging from 0 to 100, where 100 represents peak search interest for a given term within the specified time period and geography. Lower values reflect proportionally lower interest relative to that peak. RSVs are not directly comparable across different search queries.

**Table 1.** Relative search volumes (RSVs) for cosmetic procedures paired with “med spa” versus “dermatologist” queries.

Procedure type	“Med spa” RSV, mean (SD)	“Dermatologist” RSV, mean (SD)	Effect size (95% CI)^a^	*P* value	Query with greater RSV
Laser hair removal	71.1 (13.7)	63.7 (12.0)	7.39 (5.19 to 9.58)	<.001	Med spa
Botox injection	49.4 (24.5)	29.1 (6.2)	20.3 (16.5 to 24.0)	<.001	Med spa
Lip filler	35.7 (20.4)	9.10 (6.6)	26.6 (22.7 to 30.4)	<.001	Med spa
Microneedling	32.6 (25.3)	28.5 (15.8)	4.05 (1.32 to 6.78)	.004	Med spa
Laser skin resurfacing	8.89 (12.2)	48.0 (12.8)	−39.1 (−42.2 to −35.9)	<.001	Dermatologist
Microdermabrasion	18.9 (13.3)	51.7 (22.9)	−32.8 (−37.0 to −28.7)	<.001	Dermatologist
Chemical peel	22.8 (13.2)	71.6 (14.8)	−48.8 (−51.8 to −45.7)	<.001	Dermatologist

a Effect size = mean RSV (”med spa”) – mean RSV (”dermatologist”); a negative value indicates that ”dermatologist” queries had higher RSVs than ”med spa” queries.

## Discussion

Our findings suggest a substantial shift in public interest toward med spas over board-certified dermatologists for certain popular cosmetic procedures. Although increased RSVs do not necessarily equate to higher procedure volumes at med spas, prior analyses demonstrated reliable correlations between search trends and real-world demand for botulinum toxin injections and hyaluronic acid fillers [2]. Seasonality in “sunscreen” search trends has further demonstrated GT’s utility as a surrogate marker for population-level behaviors relevant to public health campaigns [3]. Together, these examples support the utility of search data as early signals of shifting patient preferences and potential emerging risks. Notably, even microneedling, which showed the smallest effect size among med spa–favoring procedures, reached statistical significance, suggesting broad rather than selective shifts in public preferences.

These trends are especially concerning, given persistent gaps in med spa regulatory oversight. Only 6.5% of surveyed US med spas reported on-site medical directorship, and fewer than half of listed directors had training in dermatology or plastic surgery [4]. State regulations governing med spa ownership, physician oversight, and nonphysicians’ scope of practice remain highly variable [5]. Cosmetic procedures performed by nonphysician operators have been associated with higher rates of adverse events when compared to physician-performed treatments (73.3% vs 51.8%), yet over 70% of patients believe nonphysician providers are adequately qualified [5]. Earlier surveys similarly linked complications from laser and resurfacing procedures to nonphysician operators, with dermatologists frequently managing the resulting adverse events [6]. As access to cosmetic services expands outside traditional medical settings, the risk of harm may increase.

This study has several limitations inherent to GT data. RSVs are normalized within regions and reflect relative Google search interest rather than absolute public demand, precluding direct comparisons across countries. GT may also underrepresent low-volume but clinically meaningful terms, and sampled results can vary across repeated queries. In a systematic review, only 7% of GT-based health studies reported sufficient methodological detail for replication [7]. RSVs may also be influenced by seasonality, as dermatologic search topics have been shown to peak during warm months, and by media-driven events producing short-lived spikes in search volumes [8,9]. Such variation could affect the apparent magnitude of differences between “med spa” and “dermatologist” queries. GT data do not account for demographic differences in internet use, may overrepresent younger populations, and do not capture data from other search engines [6,9,10].

Public search interest in med spas now outpaces that in dermatologists for several in-demand cosmetic procedures, highlighting a growing disconnect between patient perceptions and professional concerns about training and oversight. The increasing online visibility of med spas underscores the need for stronger patient and policy education regarding provider qualifications and procedural safety. Dermatologists and professional societies should prioritize accessible, evidence-based resources that help patients distinguish board-certified providers from nonphysician practitioners and understand the risks of inadequate oversight. Our findings also support ongoing advocacy for standardized credentialing requirements, physician oversight of injectable and laser-based procedures, and transparent adverse event reporting. Future research should integrate clinical outcome data, including complication rates from dermatology practices and emergency departments, with search trend analyses to more fully quantify the impact of these shifting patterns on patient safety and to guide evidence-based health policy.

## Supplementary material

10.2196/83509 Multimedia Appendix 1 Search terms used in analyses.

## Abbreviations

GT: Google Trends
HIPAA: Health Insurance Portability and Accountability Act
med spa: medical spa
RSV: relative search volume
