# Supplementary material for: Shifting Perceptions of Cosmetic Procedures: Rise of Medical Spas in Online Search Trends
Source: JMIR Dermatol. 2026 Jul 15;9:e83509. doi: 10.2196/83509 (PMC13372071; doi:10.2196/83509)
Supplement: Multimedia Appendix 1 [file derma-v9-e83509-s001.docx]

SUPPLEMENTARY APPENDIX

Table of Contents

1. Supplementary Table S1 ...............................................................................Pg. 2

**Supplementary Table S1**: Search Terms Used in Analyses

| Procedure Type | Botox | Chemical peel | Microdermabrasion | Laser skin resurfacing |
| --- | --- | --- | --- | --- |
| Search Terms | Botox® | Chemical peel | Microdermabrasion | Laser skin resurfacing |
|  | Botox® injections | Chemical skin peel | Dermabrasion | Acne scar treatment |
|  |  | Skin peel |  | Fraxel® |
| Procedure Type | Laser hair removal | Microneedling | Lip filler (collagen, fat, hyaluronic acid) |  |
| Search Terms | Laser hair removal | Microneedling | Lip filler |  |
|  | Hair removal | Dermaroller | Juvederm® |  |
|  |  | Microneedling facial |  |  |

Data Source: Google Trends ([www.google.com/trends](https://www.google.com/trends)).
